# MIDAS: software for analysis and visualisation of interallelic disequilibrium between multiallelic markers

**DOI:** 10.1186/1471-2105-7-227

**Published:** 2006-04-27

**Authors:** Tom R Gaunt, Santiago Rodriguez, Carlos Zapata, Ian NM Day

**Affiliations:** 1 Human Genetics Division, University of Southampton, School of Medicine, Duthie Building (MP 808), Southampton General Hospital, Tremona Road, Southampton SO16 6YD, UK; 2 Departamento de Genética, Universidad de Santiago, Santiago de Compostela, Spain

## Abstract

**Background:**

Various software tools are available for the display of pairwise linkage disequilibrium across multiple single nucleotide polymorphisms. The HapMap project also presents these graphics within their website. However, these approaches are limited in their use of data from multiallelic markers and provide limited information in a graphical form.

**Results:**

We have developed a software package (MIDAS – Multiallelic Interallelic Disequilibrium Analysis Software) for the estimation and graphical display of interallelic linkage disequilibrium. Linkage disequilibrium is analysed for each allelic combination (of one allele from each of two loci), between all pairwise combinations of any type of multiallelic loci in a contig (or any set) of many loci (including single nucleotide polymorphisms, microsatellites, minisatellites and haplotypes). Data are presented graphically in a novel and informative way, and can also be exported in tabular form for other analyses. This approach facilitates visualisation of patterns of linkage disequilibrium across genomic regions, analysis of the relationships between different alleles of multiallelic markers and inferences about patterns of evolution and selection.

**Conclusion:**

MIDAS is a linkage disequilibrium analysis program with a comprehensive graphical user interface providing novel views of patterns of linkage disequilibrium between all types of multiallelic and biallelic markers.

**Availability:**

Available from  and

## Background

Gametic disequilibrium (widely known as linkage disequilibrium or LD) is a genetic phenomenon which occurs when alleles at different loci are non-randomly associated in a given population. This correlation between polymorphisms is caused and/or influenced by their shared history of mutation and recombination, and by many other factors including genetic drift, population growth, admixture or migration, population structure, the ages of the polymorphisms, the physical distance separating them and the effects of selective pressure [1]. The characterization of LD is an important issue in both evolutionary and medical genetics, since it is informative in association mapping of trait or disease loci, and an indicator of the interaction between genes, the relative influence of different evolutionary forces in the generation/disruption of genetic variability, and the genetic history of populations [2].

The theory of estimation of LD has been substantially developed in recent years. Relevant advances have been made in the knowledge of the properties of LD coefficients and LD statistical tests, which are used respectively to measure the magnitude and to estimate the significance of LD. LD is said to exist when the frequency of a haplotype observed in a population sample is significantly greater or lesser than the frequency expected from the product of the allele frequencies, the magnitude of LD correlating with such difference. There are a variety of measures and statistical tests available for the estimation of LD (D', ρ, r, r^2^, d, d^2^, and chi-square and Fisher exact tests, being the most used LD coefficients and statistical tests), and many programs exist for that purpose (including Haploview, 2LD, Arlequin, GDA, DNAsp, ALLASS, DISEQ, DMAP, etc., reviewed in [3] and [4]). Some software, such as GOLD [5], GOLDsurfer [6] and Haploview [7], also include graphical displays enabling quick overviews of large regions. However, most packages are intended for use with single-nucleotide polymorphism (SNP) data in a pairwise fashion. This focus on biallelic markers makes both LD estimation and graphical representation straightforward compared with multiallelic markers such as microsatellites.

The analysis of LD between a pair of multiallelic loci represents a conceptual difference in relation to the analysis of LD between a pair of biallelic loci. In both instances, LD can be analysed at two different levels. One is the overall LD between the pair of loci, and the other is the interallelic LD between each of the alleles at the first locus and each of the alleles at the second one.

The magnitude and the significance of both overall and interallelic LD are the same for pairwise analyses involving two biallelic loci. This does not apply, however, for LD between multiallelic loci. Given a pair of multiallelic loci with *k *and *l *alleles respectively, there are *k *× *l *possible interallelic associations. In theory, pairwise combinations of alleles at different loci can differ in parameters such as magnitude, significance and patterns of LD. This has been confirmed empirically in the characterization of interallelic LD between pairs of dinucleotide repeat loci spanning human chromosome 11p15 [2], and in the analysis of LD between the *TH01 *microsatellite and *IGF2 *SNP haplotypes in the context of the identification of microsatellite loci tagging haplotypes relevant to association mapping of complex disease traits [8,9]. The analysis of interallelic associations is therefore necessary for a complete description of LD between multiallelic loci [2].

Despite the existence of alternative estimation theory [2,10,11], LD between multiallelic loci is often estimated by pooling alleles into two groups in order to reduce the system to a two-allele two-locus model. This approach does not allow the analysis of all possible interallelic associations. In contrast, it reduces the LD between multiallelic loci to a single estimate of overall LD. It has been shown that the overall measure obtained by pooling alleles of multiallelic loci tends to underestimate LD, may complicate discrimination among the evolutionary forces generating LD in populations, and may decrease the success of association mapping of trait or disease loci ([12] and references therein). In addition to the number of alleles, the magnitude and the power to detect LD depend on other factors, including the sample size, the statistical tests and coefficients used, the allele frequencies and the sign of the association [12]. This latter issue has been shown to be of special importance. A sign-based LD estimation method recently developed for multiallelic systems [2], has been shown to considerably increase both the statistical power and the accuracy of estimation of the intensity of LD [2,13]. On the other hand, the task of presenting a graphical overview of interallelic disequilibrium between alleles of multiallelic markers is rather more challenging than for biallelic markers (with colour intensity indicating the magnitude of linkage disequilibrium between a pair of markers) and has not been previously attempted.

In this work, we have developed an integrated LD analysis software (MIDAS: Multiallelic Interallelic Disequilibrium Analysis Software) that computes interallelic LD from genotypic data incorporating the latest advances in the theory of estimation of LD, and represents graphically the intensity and significance of pairwise non-random associations between any combination of microsatellites, SNPs, haplotypes or other multi-allelic markers.

## Implementation

MIDAS was written in the Python programming language v2.4 [14], using the Tkinter module for generating a graphical user interface (GUI). The Tkinter "Canvas" widget was used for plotting of graphical data, whilst other Tkinter widgets were used for creation of menus, buttons and other aspects of the interface. All modules used were part of the standard Python distribution [14] and include: Tkinter, tkFileDialog, math, copy, cPickle, os and webbrowser. The program reads and writes standard tab-delimited text files, and has an additional option to save a binary analysis file (using the cPickle module) which stores all variables and allows the user to reload a previous analysis.

**Figure 1:**
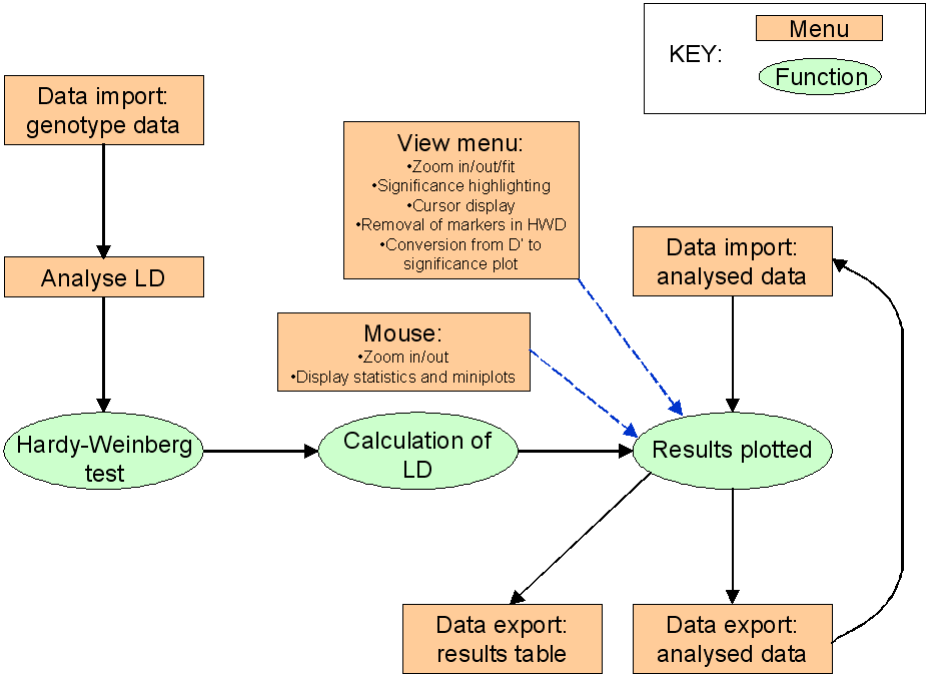
Flow-chart of MIDAS from the users perspective. Rectangles indicate user inputs, ovals indicate program functions.

**Figure 2:**
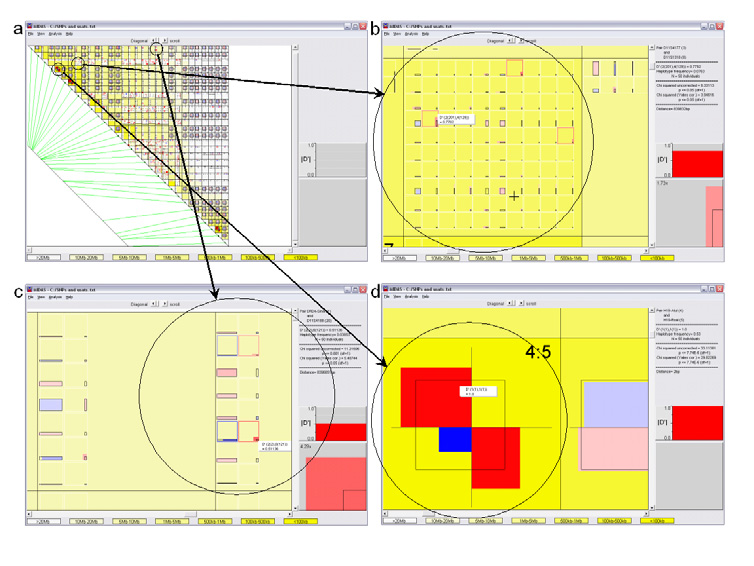
Screenshots of MIDAS. (a) A region of chromosome 11 showing 30 markers. Green lines indicate relative position of markers. Yellow intensity indicates distance between pairwise markers. Placing the mouse over a feature provides details. (b) A pairwise plot for two microsatellites (zoomed in). Significant results are boxed in red (D' ≥ 0) or blue (D'<0). Placing the mouse cursor over an allele pair provides details and statistics and also plots that pair at the bottom right of the screen. Magnitude of |D'| is also plotted (middle right). (c) A SNP/microsatellite pair (zoomed in). This is identical to the microsatellite/microsatellite plot, but with only two alleles in one dimension. (d) A SNP/SNP pair (zoomed in). The plot is oriented to place the most frequent alleles for both SNPs in the top left. Statistics can be observed by placing the mouse over an allele pair. For SNPs a magnified plot is not shown, but the |D'| graph is still used (middle right).

Figure 1 shows a flow-chart representing the program structure. Data (raw genotypes, with marker IDs and positions) are imported, and then the user selects analysis (this is a separate step to enable incorporation of different analyses in future versions). Analysis begins with an assessment of Hardy-Weinberg equilibrium (HWE) as previously described [15,16]. Markers out of HWE are flagged for highlighting in the final outputs. The next step is estimation of LD. Finally, the results of the analysis are plotted (figure 2).

The program has been designed for simple installation and use by any computer user, and requires only the prior installation of the standard Python distribution [14] to function on a Microsoft^® ^Windows^® ^2000/XP computer. Operation is mouse and menu-driven with optional hotkeys for scroll and zoom. Input files can be prepared in most spreadsheet programs and exported as tab-text. Results output is tab-text format and can be imported into most spreadsheet programs.

All parts of the program were scripted *de novo*, but the algorithm for LD calculation was based on previous programs developed by two of the authors (CZ and SR).

### Estimation of LD

Given two multiallelic loci, *A *and *B*, we estimated the LD for each pair of alleles defining a two-locus haplotype. The accurate computation of all possible interallelic associations requires that each of the two-locus haplotypes defining an interallelic combination represents only the observed and expected counts for the pair of alleles under consideration. This is not attained when alleles (and therefore haplotype counts) are pooled arbitrarily. MIDAS computes interallelic disequilibrium between multiallelic loci following an approach previously described [2] which avoids both losing and pooling interallelic information. Both this approach and its underlying theory have been applied and discussed in detail previously ([2] and references therein). In brief, if locus *A *has *k *alleles *A*_*i *_(*i *= 1,......, *k*) and locus *B *has *l *alleles *B*_*j *_(*j *= 1,......, *l*), then the complete array of possible two-locus haplotypes was partitioned into *k *× *l *separate 2 × 2 contingency tables by collapsing the data into *A*_*i *_vs. not-*A*_*i *_(Ai¯
 MathType@MTEF@5@5@+=feaafiart1ev1aaatCvAUfKttLearuWrP9MDH5MBPbIqV92AaeXatLxBI9gBaebbnrfifHhDYfgasaacH8akY=wiFfYdH8Gipec8Eeeu0xXdbba9frFj0=OqFfea0dXdd9vqai=hGuQ8kuc9pgc9s8qqaq=dirpe0xb9q8qiLsFr0=vr0=vr0dc8meaabaqaciaacaGaaeqabaqabeGadaaakeaacqWGbbqqdaWgaaWcbaGafmyAaKMbaebaaeqaaaaa@2F56@) at the *A *locus, and *B*_*j *_vs. not-*B*_*j *_(Bj¯
 MathType@MTEF@5@5@+=feaafiart1ev1aaatCvAUfKttLearuWrP9MDH5MBPbIqV92AaeXatLxBI9gBaebbnrfifHhDYfgasaacH8akY=wiFfYdH8Gipec8Eeeu0xXdbba9frFj0=OqFfea0dXdd9vqai=hGuQ8kuc9pgc9s8qqaq=dirpe0xb9q8qiLsFr0=vr0=vr0dc8meaabaqaciaacaGaaeqabaqabeGadaaakeaacqWGcbGqdaWgaaWcbaGafmOAaOMbaebaaeqaaaaa@2F5A@) at the *B *locus. Estimates of two-locus haplotype frequencies were obtained from genotype data by the Hill method, an expectation-maximisation (EM) algorithm [17]. The magnitude of disequilibrium between pairs of alleles at different loci was measured by D′ij
 MathType@MTEF@5@5@+=feaafiart1ev1aaatCvAUfKttLearuWrP9MDH5MBPbIqV92AaeXatLxBI9gBaebbnrfifHhDYfgasaacH8akY=wiFfYdH8Gipec8Eeeu0xXdbba9frFj0=OqFfea0dXdd9vqai=hGuQ8kuc9pgc9s8qqaq=dirpe0xb9q8qiLsFr0=vr0=vr0dc8meaabaqaciaacaGaaeqabaqabeGadaaakeaacuWGebargaqbamaaBaaaleaacqWGPbqAcqWGQbGAaeqaaaaa@30AD@ = *D*_*ij*_/*D*_*max*_, where *D*_*ij *_= *X*_*ij *_- *p*_*i*_*q*_*j*_, *p*_*i *_and *q*_*j *_are the frequencies of alleles *i *and *j*, respectively, *X*_*ij *_is the observed frequency of the haplotype *A*_*i*_*B*_*j *_and *D*_*max *_= min [*p*_*i*_(1 - *q*_*j*_),(1 - *p*_*i*_)*q*_*j*_] when *D*_*ij *_> 0 or *D*_*max *_= min [*p*_*i*_*q*_*j*_,(1 - *p*_*i*_)(1 - *q*_*j*_)] when *D*_*ij *_< 0 [10,18,19]. Significance test of the null hypothesis of random association between pairs of alleles at the two loci (*D*_*ij *_= 0) was tested by Xij2=nDij2/pi(1−pi)qj(1−qj)
 MathType@MTEF@5@5@+=feaafiart1ev1aaatCvAUfKttLearuWrP9MDH5MBPbIqV92AaeXatLxBI9gBaebbnrfifHhDYfgasaacH8akY=wiFfYdH8Gipec8Eeeu0xXdbba9frFj0=OqFfea0dXdd9vqai=hGuQ8kuc9pgc9s8qqaq=dirpe0xb9q8qiLsFr0=vr0=vr0dc8meaabaqaciaacaGaaeqabaqabeGadaaakeaacqWGybawdaqhaaWcbaGaemyAaKMaemOAaOgabaGaeGOmaidaaOGaeyypa0JaemOBa4Maemiraq0aa0baaSqaaiabdMgaPjabdQgaQbqaaiabikdaYaaakiabc+caViabdchaWnaaBaaaleaacqWGPbqAaeqaaOGaeiikaGIaeGymaeJaeyOeI0IaemiCaa3aaSbaaSqaaiabdMgaPbqabaGccqGGPaqkcqWGXbqCdaWgaaWcbaGaemOAaOgabeaakiabcIcaOiabigdaXiabgkHiTiabdghaXnaaBaaaleaacqWGQbGAaeqaaOGaeiykaKcaaa@4D17@, which approximates a χ^2 ^distribution with one degree of freedom, where *n *is the number of individuals sampled [20,21]. Yates's correction was also computed.

Estimation of the magnitude and significance of pairwise LD involving biallelic loci was performed in the same way, but considering that *p*_*i *_and *q*_*j *_are the frequencies of the commonest alleles for each biallelic locus. This establishes a homogeneous criterion for the construction of 2 × 2 contingency tables, (i.e., consideration of haplotype *A*_*i*_*B*_*j *_as the one constituted by the two more frequent alleles). This criterion was uniformly followed for the estimation of the observed haplotype frequency and for computation of pairwise LD magnitude and significance in all SNP/SNP analyses. This criterion is consistent with respect to the sign-based LD estimation method recently developed for multiallelic systems [2,13] and establishes consistency of some biological basis. By placing the most frequent allele of each of a pair of SNPs in the top left of the 2 × 2 table then if the minor alleles coincide on some haplotype, the display shows a 'main diagonal' excess (D' positive) whereas if minor allele at locus A predominate with major allele of locus B (and vice versa) the display shows a minor diagonal pattern (D' negative). When |D'| = 1, the haplotype patterns depicted (either three or two of the possible four) give information which is relevant to their possible history not fully evident from D' nor from r^2 ^nor any other coefficient (see figure 5).

For pairwise analyses involving two multiallelic loci, haplotype *A*_*i*_*B*_*j *_was considered to comprise the two alleles of interest. This is also consistent with the sign-based LD estimation method [2,13] in most situations, except in rare circumstances when haplotype *A*_*i*_*B*_*j *_is constituted by one allele with frequency higher than 0.5 and another allele with frequency lower than 0.5. For pairwise analyses involving one multiallelic locus and one biallelic locus, both LD estimation and representation were performed twice for each microsatellite allele of interest: *A*_*i*_*B*_*j *_was considered to comprise the microsatellite allele of interest and the commonest allele at the biallelic locus in one analysis, and the microsatellite allele of interest and the rarest allele at the biallelic locus in the other.

For users that wish to perform the analysis of multiallelic markers by dichotomising the marker into most common allele versus all other alleles combined we provide data in the output file to indicate that analysis for each combination of markers. This is provided in rows where there is a "Y" for "MostFreq1" (first marker) and "Y" for "MostFreq2" (second marker). For users who wish to collapse multiallelic markers to biallelic markers in other ways the software will accept that data in the form of input files with multiallelic markers recoded as if they were SNPs. However, it should be noted that no dichotomization represents the actual overall LD between two multiallelic loci, but only one of the possible interallelic associations.

The MIDAS output file provides D', r^2^, expected and estimated haplotype frequencies, allele frequencies, χ^2 ^and distance between markers.

## Results and discussion

An example dataset is shown in figure 2, comprising a set of microsatellites and SNPs from the 11p chromosome region [2] and Zapata *et al *(in prep) (subset of 50 samples). Figure 2a shows the typical unzoomed view, with pairs of markers in a grid of 1 to n columns and 1 to n rows. The plot begins at top left with 1 versus 2 and continues to n-1 versus n at bottom right. The distance between markers is represented by the intensity of background colour (closer = darker yellow). The image can be zoomed and scrolled, and positioning the mouse over a plotted result gives the statistics, a magnified plot and a plot of |D'| (as shown in the right-hand panel of figure 2b–d).

**Figure 3:**
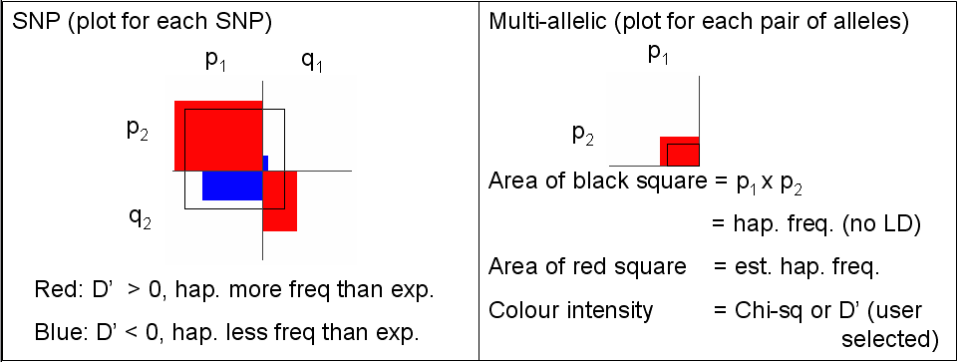
Representation of haplotype frequencies and LD in MIDAS. For SNPs the expected (under no LD) haplotype frequencies for each allele combination are plotted with an unfilled, black rectangle divided into four quadrants by two lines. The estimated haplotype frequencies are represented by solid red or blue rectangles. Where a coloured rectangle exceeds the size of the black rectangle it is coloured red, indicating an excess of that haplotype (D' ≥ 0). The opposite situation is indicated by a blue rectangle (D'<0). For multi-allelic markers the principle is the same, but a separate plot is shown for each combination of alleles, i.e. locus 1 allele i/allele not-i, locus 2 allele j/allele not-j.

For pairwise SNP analyses the LD is represented as in figure 2d. The vertical and horizontal lines split the black square into four rectangles, the areas of which represent the expected haplotype frequencies for each allele combination (upper left is *A*_*i*_*B*_*j *_frequency = *A*_*i *_× *B*_*j*_) (figure 3). Each quadrant then has a coloured rectangle to represent the "observed" (i.e. estimated using EM) haplotype frequency. The dimensions of the rectangle are in proportion to the two allele frequencies it represents, and its colour intensity represents the significance (by χ^2^) of LD or the magnitude of D' (user option on the View menu, figures in this paper show use of the significance option). Blue rectangles represent a less frequent haplotype than expected (D' < 0), and red a more frequent haplotype than expected (D' > 0). Alleles are re-ordered to ensure that the most common alleles for each marker are represented by the top-left quadrant.

For multiallelic versus biallelic or multiallelic the plot is slightly different (figure 2c). For each marker combination there are multiple pairs of vertical and horizontal lines (matching the upper left quadrant of the biallelic display). Each pair represents one allele combination, with the black rectangle indicating the expected haplotype frequency and the coloured rectangle the "observed" (i.e. estimated using EM) haplotype frequency (figure 3). The colour scheme is the same as for biallelic markers.

The markers are arrayed with marker 1 versus marker 2 at top left and marker n-1 versus marker n at bottom right, forming a right-angle triangle of plots (figure 2a). To the bottom left of the display is a line parallel to the long side of this triangle representing a map of the genomic region in which the markers are situated. Each marker is represented by a green line from their relative position on this map to the row and column in which they are plotted (figure 2a). Placing the mouse over this line (or the circle at its end) gives marker name and position.

**Figure 4:**
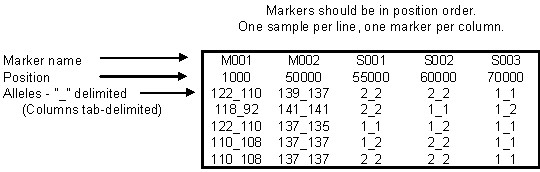
The format of a MIDAS input file. Data are raw genotypes in a tab-delimited text file. Row 1 contains marker names, row 2 contains positions. Markers should be sorted in position order for clarity. Alleles should be delimited by an underscore ("_"), and can be any valid letter or number. Where numbers are used, ensure that the same number of digits are used for all alleles (eg 094, 098, 102) to preserve size order in the alphanumeric sort. There must be no more than one blank line at the end of the data and all null values must be coded as "?_?".

A typical session involves preparation of an input file of genotypes (figure 4) using any mainstream spreadsheet program and exporting as tab-text format. MIDAS is then run by double-clicking the script file. The window shows basic instructions – briefly: (1) "Open genotype file" from "Open" on the "File" menu, then (2) Select "Analysis" – "LD and haplotypes". Zoom can be operated by mouse-click, key-stroke ("i" and "o") or menu, while scrolling can be operated by cursor key or scroll-bar. At minimum zoom ("View" – "fit to screen" eg figure 2a) the user can rapidly spot statistically significant results and patterns. Placing the mouse cursor over a feature displays its statistics and detail. The high levels of zoom shown in figures 2b,2c and 2d enable the user to analyse the LD in more detail, and read the statistics by using mouse-over. Export functions include a tab-text file of all results (which can be opened by any mainstream spreadsheet program) and a binary file format that stores all analysis variables and can be used to store the whole analysis for future use. The latter format speeds up viewing of previous analyses with regions containing many markers. Finally a postscript export option is available to save graphical view, although standard screen captures (using Alt-PrintScreen in Microsoft^® ^Windows^®^) are adequate for most uses.

Menu options include file operations, analysis, options to customise the view and a help option, which provides simple information and instructions for usage.

### Application of MIDAS

#### Evolutionary relationships between SNPs

**Figure 5:**
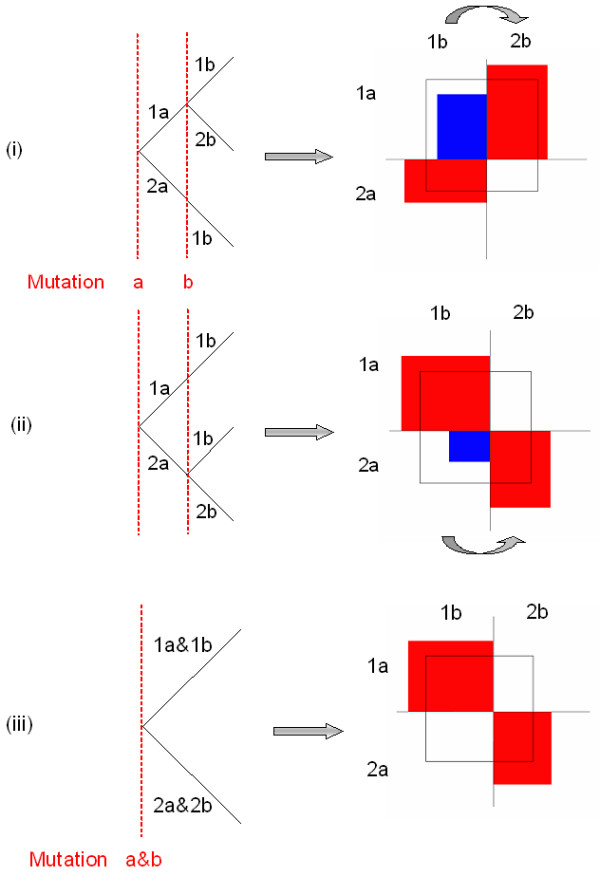
Use of MIDAS SNP/SNP plots to infer evolutionary history. The haplotype on which a SNP first arose is indicated by the estimated frequency of the haplotype carrying the most frequent alleles at both loci. (i) If this is less than expected, it implies that the SNP 2 arose on the haplotype carrying the common allele at SNP 1 (i.e. D'<0). (ii) If it is more common than expected then SNP 2 arose on the haplotype carrying the rare allele at SNP 1 (i.e. D' ≥ 0). (iii) If only two haplotypes are observed then perfect LD exists (r^2 ^= 1). This may arise through bottlenecks, selection or simultaneous occurrence.

The SNP/SNP plots (which can be seen at low levels of zoom) provide a quick way of inferring evolutionary relationships between markers. Figure 5 shows how three different type of plot provide this type of information.

#### LD between a multiallelic microsatellite and several SNPs

**Figure 6:**
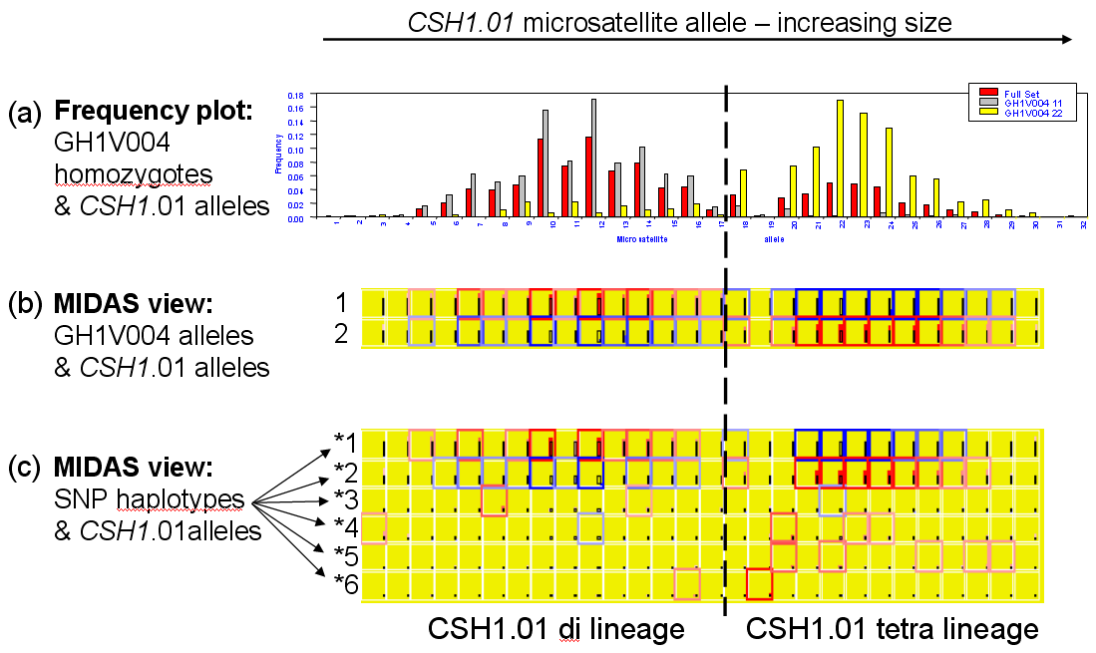
LD between a complex microsatellite and SNPs. (a) Previous work [22] indicated SNP alleles in LD with two size ranges of the *CSH1*.01 microsatellite. The lower size range has dinucleotide spacing, the upper has tetranucleotide spacing. This suggested two major lineages. (b) Plotting interallelic LD between a SNP (GH1V004) and the *CSH1*.01 microsatellite demonstrates clear LD with the two lineages. The common SNP alleles associate with the lower size range and the rare SNP alleles associate with the upper size range. Results are boxed in red where the haplotype frequency is significantly higher than expected (D' ≥ 0) and blue where it is significantly lower (D'<0). (c) SNP haplotypes (four SNPs, including GH1V004) confirm these findings and demonstrate the ability of MIDAS to handle haplotype data as a multi-allelic marker.

We have previously described the association between allele groups of a highly polymorphic microsatellite in the Growth Hormone/chorionic somatomammotrophin (*GH*/*CSH*) gene region on chromosome 17 (*CSH1*.01) and phenotypes of the metabolic syndrome [22]. For these analyses we dichotomised the microsatellite on the basis of size and distribution [22]. Figure 6 shows the interallelic LD between SNPs in the *GH*/*CSH *gene region and the *CSH1*.01 microsatellite. These analyses confirm the validity of our dichotomisation, indicating two major clades of alleles within the microsatellite. However, in most cases the analysis of all alleles of a multiallelic marker rather than a dichotomisation of alleles provides the maximum information, with no necessity to make biological assumptions.

#### LD between input haplotypes and other markers

Figure 6 also demonstrates the potential to input haplotype data and analyse it as a multi-allelic marker. In this case a 4-SNP haplotype is analysed for LD with a multi-allelic microsatellite (figure 6f). This approach can provide an overview of how biallelic or multiallelic markers interact with haplotypes, and also how haplotypes in two different regions interact with each other.

#### LD between two multiallelic tandem repeat loci

**Figure 7:**
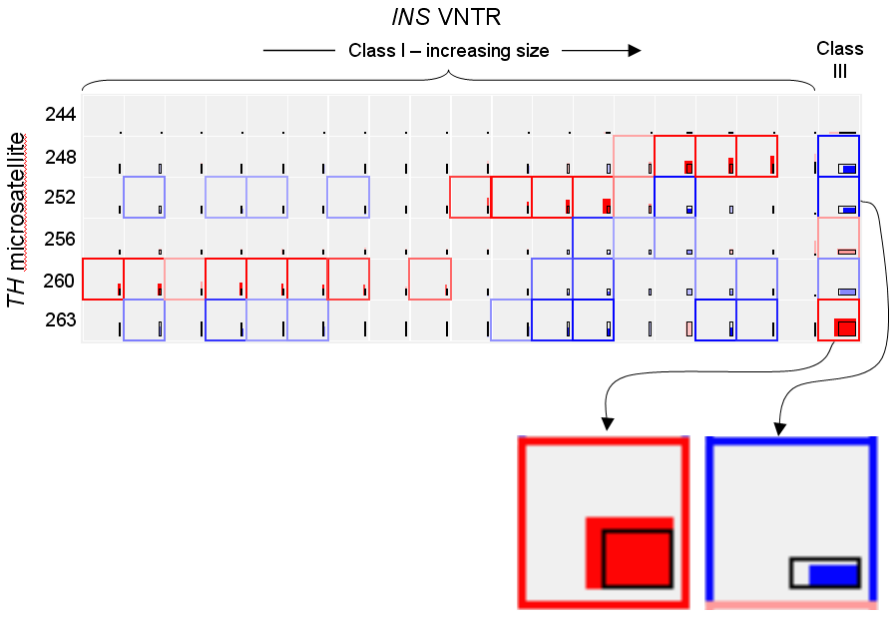
LD between the *INS *VNTR and the *TH*01 microsatellite. Each *TH*01 allele associates with a size range of VNTR alleles (256 and 263 associate with the class III alleles). This infers a greater rate of mutation in the VNTR because there is a wider range of allele sizes in the VNTR dimension significantly associated with *TH*01 alleles than *vice versa*. Close-ups of individual allele plots are shown to indicate the magnitude of effect – black rectangle indicates expected haplotype frequency under no LD, coloured rectangle indicates the estimated haplotype frequency.

Our work in the *IGF2*-*INS*-*TH *region of chromosome 11 has identified associations between SNPs, haplotypes and other markers and obesity [23-28]. Two multi-allelic markers within this region (included in our haplotype analyses) are the insulin gene VNTR (*INS *VNTR) and the tyrosine hydroxylase tetranucleotide microsatellite (*TH*01). Figure 7 shows the interallelic linkage disequilibrium between these two markers, with the patterns indicating which alleles are associated and also suggesting that the VNTR mutates more rapidly than the microsatellite (one *TH*01 allele associates with multiple *INS *VNTR alleles).

#### Regions of "perfect" LD

**Figure 8:**
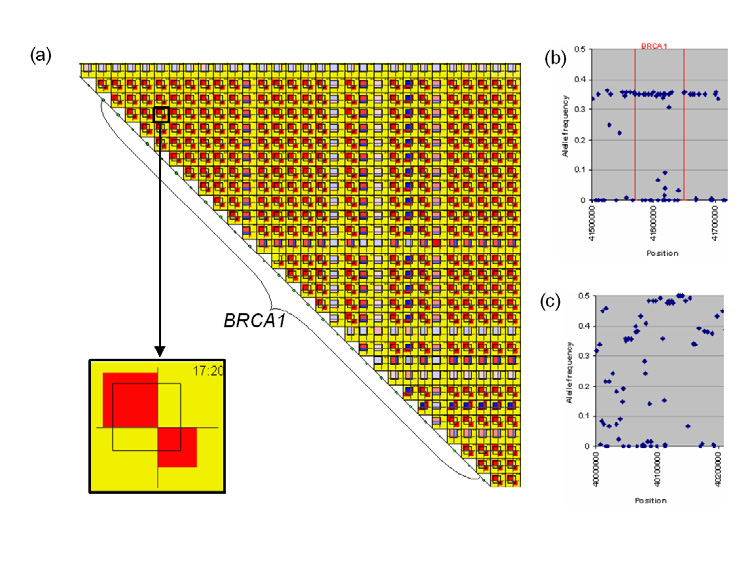
Visualisation of regions of perfect LD. Marker pairs can have either two or three haplotypes present when D' = 1. Most programs do not distinguish between these graphically, despite the potential biological importance. (a) The *BRCA1 *region on chromosome 17. MIDAS shows only two haplotypes for many SNPs (perfect LD, r^2 ^= 1) using HapMap data [29,30]. (b) Allele frequencies from HapMap data [29,30] show that SNPs in regions with only two haplotypes share the same minor allele frequency (MAF) for many SNPs (eg *BRCA1 *region on chromosome 17) compared to (c) nearby regions which have a mixture of MAFs. Viewing MAF may therefore be a quick way to find regions of perfect LD, which can then be checked with MIDAS.

Recent work using data from HapMap [29,30] and Celera has indicated the presence of extended regions of perfect LD (where only two major haplotypes exist) [31]. Figure 8a shows the characteristic MIDAS pattern for this type of region, with only two haplotypes existing for each pair of SNPs (data from HapMap [29,30]). Figure 8b and 8c demonstrates an alternative approach which we have developed (SNPFrequencyViewer) to rapidly scan for these regions, in which extended regions of isofrequent SNPs correlate with regions of perfect LD (data from HapMap [29,30]). These can then be confirmed and examined in more detail using MIDAS. Admixture of two populations highly differentiated in these genomic regions is one possible explanation, selection another.

## Conclusion

MIDAS is a new program that presents the novel approach of analysing and graphically representing the interallelic linkage disequilibrium (LD) between multiple pairs of bi- and multiallelic markers. The graphical representation of LD incorporates information on expected haplotype frequency (under no LD), estimated haplotype frequency and D' or significance. Distance information and statistics are also presented in the interface. This enables rapid visual interpretation and inference of evolutionary and functional relationships between SNPs and microsatellites across large genomic regions. Applications to data-sets we have analysed previously demonstrate the effectiveness of viewing patterns in the data graphically rather than numerically.

## Availability and requirements

• Project name: MIDAS: Multiallelic Interallelic Disequilibrium Analysis Software.

• Project home page: 

• Operating system(s): Microsoft^® ^Windows^® ^2000/XP

• Programming language: Python 2.4/Tkinter

• Other requirements: Python 2.4 or later [14] must be installed before MIDAS

• License: MIDAS licence supplied with program

• Any restrictions to use by non-academics: royalty-free use allowed within terms of licence

## Authors' contributions

MIDAS was written in Python 2.4 with a Tkinter graphical interface by TRG with design suggestions and testing by all authors. Algorithms for estimating haplotype frequencies from Hill method and for LD were adapted from a BASIC code program developed by CZ (who also suggested some statistical improvements). The manuscript was drafted by TRG and SR with inputs from all authors.
